# Boundary behaviour of $\lambda$-polyharmonic functions on regular trees

**DOI:** 10.1007/s10231-020-00981-8

**Published:** 2020-04-29

**Authors:** Ecaterina Sava-Huss, Wolfgang Woess

**Affiliations:** 1 Institut für Mathematik, Universität Innsbruck, Technikerstrasse 13, 6020 Innsbruck, Austria; 2 Institut für Diskrete Mathematik, Technische Universität Graz, Steyrergasse 30, 8010 Graz, Austria

**Keywords:** Regular tree, Simple random walk, $\lambda$-polyharmonic functions, Dirichlet and Riquier problems at infinity, Fatou theorem, 31C20, 05C05, 60G50

## Abstract

This paper studies the boundary behaviour of $\lambda$-polyharmonic functions for the simple random walk operator on a regular tree, where $\lambda$ is complex and $|\lambda |> \rho$, the $\ell ^2$-spectral radius of the random walk. In particular, subject to normalisation by spherical, resp. polyspherical functions, Dirichlet and Riquier problems at infinity are solved, and a non-tangential Fatou theorem is proved.

## Introduction

A complex-valued function *f* on a Euclidean domain *D* is called *polyharmonic of order* *n*, if it satisfies $\Delta ^n f \equiv 0$, where $\Delta$ is the classical Euclidean Laplacian. The study of polyharmonic functions originates in work of the nineteenth century and is pursued very actively. Basic references are the books by Aronszajn et al. [2] and by Gazzola et al. [8].

A classical theorem of Almansi [1] says that if the domain *D* is star-like with respect to the origin, then every polyharmonic function of order *n* has a unique decomposition

$$\begin{aligned} f(z) = \sum _{k=0}^{n-1} |z|^{2k}\, h_k(z), \end{aligned}$$

where each $h_k$ is harmonic on *D*, and |*z*| is the Euclidean length of $z \in D$. In particular, if the domain is the unit disk

$$\begin{aligned}{ \mathbb {D}}= \{ z= x + {\mathfrak {i}}\,y \in {\mathbb {C}}: |z| = \sqrt{x^2 + y^2} < 1 \}, \end{aligned}$$

then thanks to a Theorem of Helgason [9], Almansi’s decomposition can be written as an integral representation over the boundary $\partial {\mathbb {D}}$ of the disk, that is, the unit circle, with respect to the *Poisson kernel* $P(z,\xi ) = (1 - |z|^2)/|\xi - z|^2 \;(z \in {\mathbb {D}},\; \xi \in \partial {\mathbb {D}})$. Namely,

1 $$\begin{aligned} f(z) = \sum _{k=0}^{n-1} \int _{\partial {\mathbb {D}}} |z|^{2k}\, P(z,\xi )\,{\mathrm{d}}\nu _k(\xi ), \end{aligned}$$

where $\nu _0, \dots , \nu _{n-1}$ are certain distributions, namely *analytic functionals* on the unit circle. For details on those functionals, see, e.g. the nice exposition by Eymard [7].

A smaller body of work is available on the discrete counterpart, where the Laplacian is a difference operator arising from a reversible Markov chain transition matrix on a graph. Regarding boundary integral representations comparable to (1), Cohen et al. [5] have provided such a result concerning polyharmonic functions for the simple random walk operator on a homogeneous tree. This has recently been generalised by Picardello and Woess [12] to arbitrary nearest neighbour transition operators on arbitrary trees which do not need to be locally finite: [12] provides a boundary integral representation for $\lambda$-polyharmonic functions for suitable complex $\lambda$. At this point, one of the typical tasks is to study convergence properties at the boundary for $\lambda$-harmonic and -polyharmonic functions. This is the purpose of the present paper, which provides new results of this type.

Here, we come back to the specific situation of simple random walk on the homogeneous tree *T* with degree $q+1$, where $q \ge 2$. The necessary preliminaries are outlined in Sect. 2. For the transition operator *P* of the simple random walk on *T*, we study in more detail the boundary behaviour of $\lambda$-polyharmonic functions, that is, $f: T \rightarrow {\mathbb {C}}$ such that $(\lambda \cdot I - P)^n f = 0$. We assume that $\lambda \in {\mathbb {C}}{\setminus } [-\rho ,\,\rho ]$, where $\rho$ is the $\ell ^2$-spectral radius of *P* and $[-\rho ,\,\rho ]$ is its $\ell ^2$-spectrum. Close to the spirit of Korányi and Picardello [11], we extend their results from $\lambda$-harmonic to $\lambda$-polyharmonic functions, and results of the abovementioned work [5] from ordinary polyharmonic functions, i.e. $\lambda =1$, to general complex $\lambda$ in the $\ell ^2$-resolvent set of *P*.

First, we consider higher order analogues of the Dirichlet problem at infinity: in the classical case $\lambda =1$, one takes any continuous function $g$ on the boundary at infinity $\partial T$ of *T* and provides a harmonic function on *T* which provides a continuous extension of $g$ to the compactification $\widehat{T} = T \cup \partial T$. It is given by the (analogue of the) Poisson transform of $g$ with respect to the Martin kernel.

However, for $\lambda$-polyharmonic functions of higher order, as well as for $\lambda$-harmonic functions with $\lambda \ne 1$, this needs an additional normalisation, in order to control the Poisson–Martin transforms with respect to the $\lambda$-Martin kernel (and its higher order versions) at infinity. The normalisation is by spherical functions and their higher order analogues, the polyspherical functions, which to our knowledge had no previous appearance in the literature. They are introduced in Sect. 3, where we also study in the necessary detail their asymptotic behaviour at infinity, see Proposition 3.5.

The first two main results are given by the “twin” Theorems 4.1 and 4.6 in Sect. 4. The (analogue of the) Poisson integral of $g$ with respect to the $n{\text {th}}$ extension of the $\lambda$-Martin kernel (i.e. the kernel multiplied by the—suitably normalised—$n{\text {th}}$ power of the Busemann function) is polyharmonic of order $n+1$, and normalised (= divided) by the $n{\text {th}}$ polyspherical function, it converges to $g$ at the boundary. Next, Theorem 4.6 concerns Fatou type non-tangential convergence of polyharmonic extensions of complex Borel measures on the boundary. While the proofs of these results follow classical lines, the main point here is that one first had to understand how to exploit the boundary integral representation of $\lambda$-polyharmonic functions and that the most natural normalisation is by the polyspherical functions. This allows to apply much less involved methods than, for example, those used in [5] for the special case $\lambda =1$.

In general, the polyharmonic extension of a continuous boundary function cannot be unique because one may add lower order polyharmonic functions that do not change the limit. However, uniqueness is proved in the case of $\lambda$-harmonic functions ($n=1$), see Theorem 4.7. That is, normalising by the associated spherical function, the solution of the $\lambda$-Dirichlet problem at infinity is unique. Note that since $\lambda$ is in general complex, typical tools from Potential Theory such as the maximum principle cannot be applied here and are replaced by a new idea, using spherical averages, which we have not encountered before in this context.

As a corollary of these results, a tree-counterpart of the *Riquier problem* at infinity is provided. In the case of a bounded Euclidean domain *D* as above, this consists in providing continuous boundary functions $g_0,\ldots , g_{n-1}$ and looking for a polyharmonic function *f* of order *n* on *D* such that $\Delta ^k f$ is a continuous extension of $g_k$ for each *k*. For finite graphs, the analogous problem has been studied in a note by Hirschler and Woess [10], where one can find further references concerning the discrete setting. In the case of $\lambda$-harmonic functions on *T*, the formulation of the analogous problem requires again suitable normalisation, see Definition 4.9 and Corollary 4.10.

## Homogeneous trees and boundary integral representations

Let $T= T_q$ be the homogeneous tree where each vertex has $q+1 \ge 3$ neighbours. We need some features of its structure and first recall the well-known boundary $\partial T$ of the tree. For $x, y \in T$, there is a unique *geodesic path* $\pi (x,y) = [x=x_0,\, x_1,\ldots , x_n=y]$ of minimal length *n*, such that $x_{k-1} \sim x_k$ for $x = 1, \ldots ,n$, and $d(x,y)=n$ is the graph distance between *x* and *y*. A *geodesic ray* is a sequence $[x_0,\, x_1,x_2,\ldots ]$ of distinct vertices with $x_{n-1} \sim x_n$. Two rays are equivalent if they share all but finitely many among their vertices. An *end* of *T* is an equivalence class of geodesic rays, and $\partial T$ is the set of all ends. For any $\xi \in \partial T$ and $x \in T$, there is a unique geodesic $\pi (x,\xi )$ which starts at *x* and represents $\xi$. Next, we choose a root vertex $o \in T$. We set $\widehat{T} = T \cup \partial T$. For any pair of points $z, w \in \widehat{T}$, their *confluent* $z \wedge w$ is the last common vertex on the finite or infinite geodesics $\pi (o,w)$ an $\pi (o,z)$, unless $z=w$ is an end, in which case $z \wedge z = z$. Furthermore, for a vertex $x \ne o$, we define its *predecessor* $x^-$ as the neighbour of *x* on the arc $\pi (o,x)$.

We now equip $\widehat{T}$ with a new metric: we set $|x| = d(x,o)$ for $x \in T$, and let

2 $$\begin{aligned} \theta (z,w) = {\left\{ \begin{array}{ll} q^{-|z \wedge w|},&{\text {if }}\; z \ne w,\\ 0,&{\text {if }}\; z = w. \end{array}\right. } \end{aligned}$$

This is an ultra-metric which turns $\widehat{T}$ into a compact space with *T* as an open, discrete and dense subset. A basis of the topology is given by all *branches* $\widehat{T}_{x,y}\,$, where $x, y \in T$ with $x \ne y$. Here,

$$\begin{aligned} \widehat{T}_{x,y} = \{ z \in \widehat{T} : y \in \pi (x,z) \}. \end{aligned}$$

This is a compact-open set, and its boundary $\partial T_{x,y} = \widehat{T}_{x,y} \cap \partial T$ is called a *boundary arc*. As a matter of fact, a basis of the topology of $\partial T$ is given by the collection of all $\partial T_x := \partial T_{o,x}\,$, including $\partial T_o := \partial T$. A *locally constant function* on $\partial T$ is a finite linear combination

$$\begin{aligned} g= \sum _{j=1}^n c_j\,{\mathbf {1}}_{\partial T_{x_j}} \end{aligned}$$

of indicator functions of boundary arcs. It can equivalently be written in terms of boundary arcs $\partial T_{x,y_k}$ for any fixed vertex *x*. A *distribution* on $\partial T$ is an element of the dual of the linear space of locally constant functions. Equivalently, it can be written as a finitely additive measure $\nu$ on the collection of all boundary arcs. For this, it suffices to consider only the boundary arcs with respect to *o*, so that $\nu$ is characterised as a set function

3 $$\begin{aligned} \nu : \{ \partial T_x : x \in T \} \rightarrow {\mathbb {C}}\quad {\text {with}}\quad \nu (\partial T_x) = \sum _{y: y^- = x} \nu (\partial T_y) \quad {\text {for all }}\;x. \end{aligned}$$

For $g$ as above, we write $\nu (g)$ as an integral

$$\begin{aligned} \int _{\partial T} g\, {\mathrm{d}}\nu = \sum _{j=1}^n c_j \, \nu (\partial T_{x_j}). \end{aligned}$$

When $\nu$ is non-negative real, compactness yields immediately that it extends to a $\sigma$-additive measure on the Borel $\sigma$-algebra of $\partial T$. In general, $\nu$ does not necessarily extend to a $\sigma$-additive complex measure; see Cohen et al. [6].

We now turn to harmonic functions. For a function $f: T \rightarrow {\mathbb {C}}$, we define

$$\begin{aligned} Pf(x) = \frac{1}{q+1} \sum _{y: y \sim x} f(y), \end{aligned}$$

where $y \sim x$ means that the vertices $x, y \in T$ are neighbours. *P* is the transition operator of the simple random walk on *T*. We recall the very well-known fact that as a self-adjoint operator on the space $\ell ^2(T)$, its spectrum is the interval $[-\rho ,\,\rho ]$, where $\rho = 2\sqrt{q}/(q+1)$. In this setting, the discrete counterpart of the Laplacian is $P-I$, where *I* is the identity operator.

### Definition 2.1

For $\lambda \in {\mathbb {C}}$, a $\lambda$-*polyharmonic function of order* *n* is a function $f:T \rightarrow {\mathbb {C}}$ such that $(\lambda \cdot I - P)^n f = 0$.

For $n=1$, it is called $\lambda$-harmonic, and when $\lambda =1$, we speak of a polyharmonic, resp. harmonic function.

Following [12], for a suitable boundary integral representation, the “eigenvalue” $\lambda$ should belong to the resolvent set ${\mathsf {res}}(P) = {\mathbb {C}}{\setminus } [-\rho ,\,\rho ]$ of *P* on $\ell ^2(T)$. In this case, let $G(x,y|\lambda ) = (\lambda \cdot I - P)^{-1} \delta _y(x)$ be the Green function, that is, the (*x*, *y*)-matrix element of the resolvent, where $x,y \in T$. By [12, Thm. 4.2], or by direct computation, $G(x,x|\lambda ) \ne 0$, and we can define $F(x,y|\lambda ) = G(x,y|\lambda )/G(x,x|\lambda )$. These functions depend only on the graph distance *d*(*x*, *y*) between *x* and *y*.

For $|\lambda | \ge \rho$, one has a combinatorial-probabilistic interpretation:

4 $$\begin{aligned} F(x,y|\lambda ) = \sum _{n=1}^{\infty } f^{(n)}(x,y)/\lambda ^n, \end{aligned}$$

where $f^{(n)}(x,y)$ is the probability that the simple random walk starting at *x* hits *y* at the $n{\text {th}}$ step for the first time. Simple and well-known computations yield

5 $$\begin{aligned} \begin{aligned} F(x,y|\lambda )&= F(\lambda )^{d(x,y)}, \quad {\text {where}}\\ F(\lambda )&= \frac{q+1}{2q} \bigl (\lambda - s(\lambda )\bigr ) \quad {\text {with}}\quad s(\lambda ) = \lambda \, \sqrt{1- \rho ^2/\lambda ^2}, \end{aligned} \end{aligned}$$

see, e.g. [13, Lemma 1.24] (with $z=1/\lambda$). The complex square root is $\sqrt{r{\mathrm{e}}^{i\phi }} = \sqrt{r}{\mathrm{e}}^{i\phi /2}$ for $\phi \in (-\pi ,\,\pi )$.

The $\lambda$-*Martin kernel* on $T \times \partial T$ is

$$\begin{aligned} K(x,\xi |\lambda ) = \frac{F(x,x\wedge \xi |\lambda )}{F(o,x \wedge \xi |\lambda )} = F(\lambda )^{{\mathfrak {h}}(x,\xi )}, \quad x \in T,\;\xi \in \partial T, \end{aligned}$$

where

$$\begin{aligned} {\mathfrak {h}}(x,\xi ) = d(x,x\wedge \xi ) -d(o, x \wedge \xi ) \end{aligned}$$

is the *Busemann function* or *horocycle index* of *x* with respect to the end $\xi$. Note that for fixed *x*, the function $\xi \mapsto K(x,\xi |\lambda )$ is locally constant.

Now, a basic result in the seminal paper of Cartier [4], valid for real $\lambda \ge \rho$, and its extension to complex $\lambda \in {\mathsf {res}}(P)$ [12] says the following for simple random walk on *T*.

For $\lambda \in {\mathbb {C}}{\setminus } [-\rho ,\,\rho ]$, every $\lambda$-harmonic function *h* on *T* has a unique integral representation

6 $$\begin{aligned} h(x) = \int _{\partial T} K(x,\xi |\lambda )\, {\mathrm{d}}\nu (\xi ), \end{aligned}$$

where $\nu$ is a distribution on $\partial T$ as in (3). If $\lambda > \rho$ and $h > 0$, then $\nu$ is a positive Borel measure. Indeed, this holds for arbitrary nearest neighbour random walks on arbitrary countable trees, and [12] has a method to extend this to a boundary integral representation of $\lambda$-polyharmonic functions. Specialised to simple random walk on $T = T_q\,$, this yields the following extension of a result of [5], where the basic case $\lambda =1$ is considered.

### **Theorem 2.2**

[12] *For *$\lambda \in {\mathbb {C}}{\setminus } [-\rho ,\,\rho ]$, * every *$\lambda$*-polyharmonic harmonic function f of order n* *on T has a unique integral representation *

$$\begin{aligned} f(x) = \sum _{k=0}^{n-1}\int _{\partial T} K(x,\xi |\lambda )\, {\mathfrak {h}}_k(x,\xi |\lambda )\, {\mathrm{d}}\nu _k(\xi ) \quad {\text {with}}\quad {\mathfrak {h}}_k(x,\xi |\lambda ) = \frac{{\mathfrak {h}}(x,\xi )^k}{k!\,s(\lambda )^k}, \end{aligned}$$

*where *$\nu _0,\ldots , \nu _{n-1}$* are distributions on *$\partial T$.

The normalisation by $k!\,s(\lambda )^k$, where $s(\lambda )$ is as in (5), is not present in [12, Cor. 5.4]. We shall see below in Lemma 3.4 why it is useful.

## Polyspherical functions

### Definition 3.1

For any $\lambda \in {\mathbb {C}}$, the *spherical function* $\Phi (x|\lambda )$ is the unique function on *T* with $\Phi (o|\lambda )=1$ which is $\lambda$-harmonic and *radial,*, i.e. it depends only on $|x|= d(o,x)$.

Namely, if we set $\varphi _k(\lambda ) = \Phi (x|\lambda )$ for $|x|=k$, then we have the recursion

$$\begin{aligned} \varphi _0(\lambda )=1,\quad \varphi _1(\lambda ) = \lambda ,\quad {\text {and}}\quad \lambda \,\varphi _k(\lambda ) = \frac{1}{q+1}\,\varphi _{k-1}(\lambda ) + \frac{q}{q+1}\,\varphi _{k+1}(\lambda ) \quad {\text {for }}\; k \ge 1. \end{aligned}$$

We shall consider the case when $\lambda$ is in the $\ell ^2$-resolvent set of *P*, that is, $\lambda \in {\mathbb {C}}{\setminus } [\rho ,\,\rho ]$. Let $F(\lambda )$ be as in (5), and let

7 $$\begin{aligned} \widetilde{F}(\lambda ) = \frac{q+1}{2q} \bigl (\lambda + s(\lambda )\bigr ) \end{aligned}$$

be the second solution, besides $F(\lambda )$, of the equation

8 $$\begin{aligned} \lambda \,F(\lambda ) = \frac{1}{q+1} + \frac{q}{q+1}F(\lambda )^2. \end{aligned}$$

Then, one can solve the above recursion, and

9 $$\begin{aligned} \begin{aligned} \Phi (x|\lambda )&= a(\lambda )\,F(\lambda )^{|x|} + \tilde{a}(\lambda )\, \widetilde{F}(\lambda )^{|x|},\quad {\text {where}}\\ a(\lambda )&= \frac{s(\lambda )-\frac{q-1}{q+1}\lambda }{2s(\lambda )} \quad {\text {and}}\quad \tilde{a}(\lambda ) = \frac{s(\lambda )+\frac{q-1}{q+1}\lambda }{2s(\lambda )}. \end{aligned} \end{aligned}$$

We collect a few elementary properties.

### **Lemma 3.2**

*We have for* $\lambda \in {\mathbb {C}}{\setminus } [\rho ,\,\rho ]$

$$\begin{aligned} 0< |F(\lambda )|< 1\big /\sqrt{q} < |\widetilde{F}(\lambda )|, \quad F(1) = 1/q, \quad {\text {and}}\quad \widetilde{F}(1)=1. \end{aligned}$$

*Furthermore,*

$$\begin{aligned} \Phi (x|1) = 1 \quad {\text {and}}\quad \Phi (x|\lambda ) \ne 0 \quad {\text {for all }}\; x \in T. \end{aligned}$$

### *Proof*

First of all, by (8), $F(\lambda ) \ne 0$ and $F(\lambda )\widetilde{F}(\lambda ) = 1/q$. Next, by (4), $|F(\lambda )| \le F(|\lambda |) < F(\rho ) = 1\big /\sqrt{q}\,$ for $|\lambda | > \rho$. Also when $|\lambda | = \rho$ and $\lambda \ne \pm \rho$, we have $|F(\lambda )| < F(\rho ) = 1\big /\sqrt{q}\,$. At last, for $\lambda$ in the real interval $(-\rho ,\rho )$, the limits of $F(\cdot )$ are

$$\begin{aligned} \frac{q+1}{2q} \bigl (\lambda \pm {\mathfrak {i}}\,\sqrt{\rho ^2 - \lambda ^2}\bigr ), \end{aligned}$$

according to whether $\lambda$ is approached within the upper or lower half plane. Thus, in the upper open semidisk $\{ z \in {\mathbb {C}}: |z| < \rho ,\; {\mathfrak {R}}z > 0 \}$, as well as in the corresponding lower open semidisk, $F(\lambda )$ is analytic, and its absolute values at the boundary are $\le 1\big /\sqrt{q}\,$. By the Maximum Modulus Principle, $|F(\lambda )| <1/\sqrt{q}\,$ within each of those two semidisks. We see that the last inequality holds in all of ${\mathbb {C}}{\setminus } [\rho ,\,\rho ]$.

Consequently, $|q \widetilde{F}(\lambda )| = 1/|F(\lambda )| > \sqrt{q}$. The values for $\lambda =1$ are obvious.

Finally, we claim that for the coefficient functions in (9) one has $|a(\lambda )| < |\tilde{a}(\lambda )|$. For $|\lambda | > \rho$, as well as for $|\lambda | = \rho$ and $\lambda \ne \pm \rho$, one can see this from the fact that $1- \rho ^2/\lambda ^2$ belongs to the complex half-plane with positive real part. For $\lambda$ in one of the above two semidisks, one can proceed as above: one checks that $\tilde{a}(\lambda ) \ne 0$. Then, the function $a(\lambda )/\tilde{a}(\lambda )$ is analytic in each semidisk, with boundary values whose absolute values are $\le 1$, and the desired inequality follows. Therefore,

$$\begin{aligned} \bigl |a(\lambda )\,F(\lambda )^{|x|}\bigr | < \bigl |\tilde{a}(\lambda )\, \widetilde{F}(\lambda )^{|x|}\bigr | \end{aligned}$$

for every $x \in T$, and $\Phi (x|\lambda ) \ne 0$.

We can describe the spherical functions via their integral representation (6). Let ${\mathsf m}$ stand for the *uniform distribution* on $\partial T$. This is the Borel probability measure which for each $k \in {\mathbb {N}}_0$ assigns equal mass to all boundary arcs $\partial T_x\,$, where $x \in T$ with $|x|=k$. That is,

$$\begin{aligned} {\mathsf m}(\partial T_x) = {\left\{ \begin{array}{ll} 1,&{\text {if }}\; x = o,\\ 1\big /\bigl ((q+1)q^{|x|-1}\bigr ), &{\text {if }}\; x \ne o. \end{array}\right. } \end{aligned}$$

We shall often write ${\mathrm{d}}{\mathsf m}(\xi ) = {\mathrm{d}}\xi \,$. Then,

10 $$\begin{aligned} \Phi (x|\lambda ) = \int _{\partial T} K(x,\xi |\lambda )\, {\mathrm{d}}\xi . \end{aligned}$$

Indeed, the right-hand side satisfies all requirements of Definition 3.1, which determine the spherical function. A comparison with Theorem 2.2 leads us to the following.

### Definition 3.3

For $n \ge 0$, the $n{\text {th}}$ *polyspherical function* is

$$\begin{aligned} \Phi _n(x|\lambda ) = \int _{\partial T} K(x,\xi |\lambda )\,{\mathfrak {h}}_n(x,\xi |\lambda ) \, {\mathrm{d}}\xi . \end{aligned}$$

It is $\lambda$-polyharmonic of order $n+1$, and it is radial. With respect to those two properties, it is uniquely determined by its values for $|x|=0, 1, \ldots , n$. For $n \ge 1$, its value at $x=o$ is 0. For $n=0$, it is of course the spherical function (10).

In particular, $(\lambda \cdot I - P)^n\, \Phi _n(\cdot |\lambda )$ is $\lambda$-harmonic and radial, so that it must be a multiple of $\Phi (\cdot |\lambda )$. In order to determine the factor, we need to recall part of how Theorem 2.2 was obtained in [12]. Let $K^{(n)}(x,\xi |\lambda )$ be the $n^{\text {th}}$ derivative of $K(x,\xi |\lambda )$ with respect to $\lambda$. Then,

11 $$\begin{aligned} \frac{(-1)^n}{n!}(\lambda \cdot I - P)^n K^{(n)}(\cdot ,\xi |\lambda ) = K(\cdot ,\xi |\lambda ). \end{aligned}$$

In [12, equation (5.2)], it is shown that

12 $$\begin{aligned} K^{(n)}(x,\xi |\lambda ) = K(x,\xi |\lambda )\, \sum _{k=1}^n {\mathfrak {h}}(x,\xi )^k \, g_{k,n}(\lambda ), \end{aligned}$$

where the functions $g_{k,n}(\lambda )$ are given recursively; in particular, with $s(\lambda )$ as in (5),

$$\begin{aligned} g_{n,n}(\lambda ) = (-1)^n s(\lambda )^{-n} . \end{aligned}$$

Combining (11) and (12), we get

### **Lemma 3.4**

$$(\lambda \cdot I - P)^n \bigl [K(\cdot ,\xi |\lambda )\, {\mathfrak {h}}_n(x,\xi |\lambda ) \bigr ] = K(\cdot ,\xi |\lambda ).$$

Integrating with respect to ${\mathrm{d}}\xi$, we also obtain the following.

13 $$\begin{aligned} (\lambda \cdot I - P)^n \Phi _n(\cdot |\lambda ) = \Phi (\cdot |\lambda ). \end{aligned}$$

We shall need the asymptotic behaviour of $\Phi _n(x|\lambda )$ as $|x| \rightarrow \infty$.

### Proposition 3.5

*Let *$\lambda \in {\mathbb {C}}$* with *$|\lambda | > \rho$*. Then, as *$|x| \rightarrow \infty$,

$$\begin{aligned} \Phi _n(x|\lambda ) \sim \tilde{a}(\lambda )\, \frac{(-1)^n\, |x|^n}{n! \, s(\lambda )^n}\, \widetilde{F}(\lambda )^{|x|}, \end{aligned}$$

*with *$\tilde{a}(\lambda )$* given by *(9)*. In particular, in the standard case *$\lambda =1$*, we have *$\widetilde{F}(1) = \tilde{a}(1) = 1$.

*Therefore, there is *$R = R_{n,\lambda } > 0$* such that*

$$\begin{aligned} \Phi _n(x|\lambda ) \ne 0 \quad {\text {and}}\quad |\Phi _n(x|\lambda )| \le 2 |\tilde{a}(\lambda )|\, \frac{|x|^n}{n! \, |s(\lambda )|^n}\, |\widetilde{F}(\lambda )|^{|x|} \quad {\text {for }}\; |x| \ge R. \end{aligned}$$

*Furthermore,*

14 $$\begin{aligned} \lim _{|x| \rightarrow \infty } \frac{\Phi _k(x|\lambda )}{\Phi _n(x|\lambda )} = 0 \quad {\text {for }}\; k < n. \end{aligned}$$

### *Proof*

By Lemma 3.2,

$$\begin{aligned} | q \, \widetilde{F}(\lambda )^2|> 1 \quad {\text {for}} \quad |\lambda | > \rho . \end{aligned}$$

Now, let $x \in T {\setminus } \{o\}$. For $\ell \in \{ 0, 1, \ldots , |x|\}$, let $A_{\ell } = \{ \xi \in \partial T : |x \wedge \xi | = \ell \}$, and set ${\mathsf m}_{\ell } = m(A_{\ell })$. Then,

$$\begin{aligned} K(x,\xi |\lambda ) = F(\lambda )^{|x|-2\ell } \; {\text { for }} \xi \in A_{\ell },\quad {\text {and}}\quad {\mathsf m}_{\ell } = {\left\{ \begin{array}{ll} \dfrac{q}{q+1}&{\text {for}}\;\ell =0,\\ \dfrac{q-1}{(q+1)q^{\ell }}&{\text {for}}\;\ell =1,\ldots , |x|-1,\\ \dfrac{1}{(q+1)q^{|x|-1}}&{\text {for}}\;\ell =|x|. \end{array}\right. } \end{aligned}$$

We use $F(\lambda ) = \bigl (q \widetilde{F}(\lambda )\bigr )^{-1}$ and set $k=|x|-\ell$. Then, the integral formula of Definition 3.3 translates into

$$\begin{aligned} \begin{aligned} n! \, s(\lambda )^n\,\Phi _n(x|\lambda )&= \sum _{\ell =0}^{|x|} F(\lambda )^{|x|-2\ell } \,\bigl (|x|-2\ell \bigr )^n\,{\mathsf m}_{\ell }\\&= \frac{q}{q+1} \, \bigl (-|x|\bigr )^n\, \widetilde{F}(\lambda )^{|x|} \left( 1 + \frac{q-1}{q}\sum _{k=1}^{|x|-1} \bigl ( q\,\widetilde{F}(\lambda )^2\bigr )^{-k} \Bigl (\frac{|x|-2k}{|x|}\Bigr )^{\!n} + (-1)^n \bigl ( q\,\widetilde{F}(\lambda )^2\bigr )^{-|x|}\right) . \end{aligned} \end{aligned}$$

The last term within the big parentheses tends to 0 as $|x| \rightarrow \infty \,$. Decompose the sum into the two pieces where in the first one, summation is over $1 \le k \le \sqrt{|x|}$ and in the second one, summation is over $k > \sqrt{|x|}$. Then, the second part is a remainder of a convergent series, so that it also tends to 0 as $|x| \rightarrow \infty$. Now, in the range $1 \le k \le \sqrt{|x|}$, the quotients $(|x|-2k)/|x|$ tend to 1 uniformly as $|x| \rightarrow \infty$. Therefore, the first part of the sum converges to

$$\begin{aligned} \frac{q-1}{q} \sum _{k=1}^{\infty } \bigl ( q\,\widetilde{F}(\lambda )\bigr )^{-k} = \frac{q-1}{q} \frac{1}{q\,\widetilde{F}(\lambda )^2-1}, \end{aligned}$$

as $|x| \rightarrow \infty$. This yields the proposed asymptotic formula, with some elementary computations for getting the factor $\tilde{a}(\lambda )$.

## Dirichlet-, Riquier- and Fatou-type convergence

In the classical case of harmonic functions, that is, when $\lambda =1$, the Dirichlet problem asks whether for any real or complex-valued function $g\in {\mathcal {C}}(\partial T)$, there is a continuous extension to $\widehat{T}$ which is harmonic in *T*. That is, we look for a function $h = h_{g}$ on *T* such that

$$\begin{aligned} (I-P)h = 0 \quad {\text {and}}\quad \lim _{x \rightarrow \xi } h(x) = g(\xi ) \quad {\text {for every }}\; \xi \in \partial T. \end{aligned}$$

If a solution exists, then it is necessarily unique by the minimum (maximum) principle. For our simple random walk on *T*, it is folklore that the Dirichlet problem is solvable, and that the solution is given as the *Poisson integral* of $g\,$:

$$\begin{aligned} h(x) = \int _{\partial T} K(x,\xi |1)\, g(\xi )\, {\mathrm{d}}\xi . \end{aligned}$$

We are now interested in the general case when $\lambda \in {\mathbb {C}}{\setminus } [-\rho ,\,\rho ]$, which will remain fixed throughout this section. First of all, the above question is not well-posed. Indeed, if for example $\lambda > 1$ is real, then the “Poisson integral” of the constant function $\mathbf {1}$ on $\partial T$ is $\Phi (x|\lambda )$. By Proposition 3.5, it tends to $\infty$ as $|x| \rightarrow \infty$, since $\widetilde{F}(\lambda ) > 1$. Thus, we need to normalise, compare with [11]. The same is necessary for the polyharmonic versions of higher order.

### **Theorem 4.1**

*Let *$\lambda \in \mathbb {C}$* with *$|\lambda | > \rho$*. For *$g\in {\mathcal {C}}(\partial T)$* and *$n \ge 0$*, set*

$$\begin{aligned} f(x) = \int _{\partial T} K(x,\xi |\lambda )\,{\mathfrak {h}}_n(x,\xi |\lambda ) \, g(\xi )\, {\mathrm{d}}\xi \end{aligned}$$

*Then, f* is $\lambda$*-polyharmonic of order *$n+1$* and *

15 $$\begin{aligned} \lim _{x \rightarrow \xi } \frac{f(x)}{\Phi _n(x|\lambda )} = g(\xi ) \quad {\text {for every }}\; \xi \in \partial T. \end{aligned}$$

Before the proof of this result, we introduce the normalized kernel

16 $$\begin{aligned} \mathcal {K}_n(x,\xi |\lambda ) = \frac{K(x,\xi |\lambda )\,{\mathfrak {h}}_n(x,\xi |\lambda )}{\Phi _n(x|\lambda )}, \quad n \ge 0. \end{aligned}$$

We only need it for large |*x*|, and then $\Phi _n(x|\lambda ) \ne 0$ by Proposition 3.5, so that the division in (15) and the definition of ${\mathcal {K}}_n$ are legitimate. If we fix such an $x \in T$ with $|x| \ge R$, the function $\xi \mapsto {\mathcal {K}}_n(x,\xi |\lambda )$ is locally constant, since it depends only on $x \wedge \xi$ which ranges within the finite geodesic $\pi (o,x)$. Therefore, it is continuous.

### **Lemma 4.2**

*Let *$$y \in T$$*. Then,*

$$\begin{aligned} \lim _{|x| \rightarrow \infty ,\, x \in \partial T_y} {\mathcal {K}}_n(x,\xi |\lambda ) = 0 \end{aligned}$$

*uniformly for *$$\xi \in \partial T {\setminus } \partial T_y\,$$.

### *Proof*

If $x \in T_y$ and $\xi \in \partial T {\setminus } \partial T_y$, then $x \wedge \xi = y \wedge \xi \in \pi (o,y)$. We have

$$\begin{aligned} {\mathfrak {h}}(x,\xi ) = |x| - 2|y \wedge \xi | \ge |x| - 2|y|. \end{aligned}$$

Therefore, using Lemma 3.2 and Proposition 3.5,

$$\begin{aligned} {\mathcal {K}}_n(x,\xi |\lambda ) \sim \frac{|F(\lambda )|^{-2|y\wedge \xi |}}{|\tilde{a}(\lambda )|} \, \left| \frac{F(\lambda )}{\widetilde{F}(\lambda )}\right| ^{|x|}\, \left( \frac{|x|-2|y\wedge \xi |}{|x|}\right) ^{n}, \end{aligned}$$

which tends to 0 as proposed.

### *Proof of Theorem 4.1*

For $$x \in T$$ with $$|x| \ge R$$,

$$\begin{aligned} {\mathrm{d}}\mu _x(\xi ) = {\mathcal {K}}_n(x,\xi |\lambda )\,{\mathrm{d}}\xi \end{aligned}$$

defines a complex Borel measure on $\partial T$. (It also depends on $\lambda$ and *n*, which we omit in the present notation.) We have $\mu _x(\partial T) = 1.$ We write $|\mu |_x$ for its total variation measure. Its density with respect to ${\mathrm{d}}\xi$ is $\bigl |K(x,\xi |\lambda )\,{\mathfrak {h}}_n(x,\xi |\lambda )\bigr |\big /\bigl |\Phi _n(x|\lambda )\bigr |$. Let us write

$$\begin{aligned} |\Phi |_n(x|\lambda ) = \int _{\partial T} \bigl |K(x,\xi |\lambda )\,{\mathfrak {h}}_n(x,\xi |\lambda )\bigr |\,{\mathrm{d}}\xi . \end{aligned}$$

A computation completely analogous to the one in the proof of Proposition 3.5 shows that

$$\begin{aligned} |\Phi |_n(x|\lambda ) \sim C(\lambda )\, \frac{|x|^n}{n!\,|s(\lambda )|^n}\,|\widetilde{F}(\lambda )|^{|x|} ,\quad {\text {where}} \quad C(\lambda ) = \frac{1}{q+1}\, \frac{q^2 |\widetilde{F}(\lambda )|^2 - 1}{q |\widetilde{F}(\lambda )|^2 - 1}. \end{aligned}$$

Therefore,

17 $$\begin{aligned} |{\mathsf m}|_x(\partial T) = \frac{|\Phi |_n(x|\lambda )}{|\Phi _n(x|\lambda )|} \rightarrow \frac{C(\lambda )}{|\tilde{a}(\lambda )|}, \quad {\text {as }}\; |x| \rightarrow \infty . \end{aligned}$$

We can now prove (15) along classical lines. Let $\xi _0 \in \partial T$ and $\varepsilon > 0$. Then, given $g\in C(\partial T)$, there is a neighbourhood of $\xi _0$ on which $|g(\xi )-g(\xi _0)|<\varepsilon$. We may assume that this neighbourhood is of the form $\partial T_y\,$, where $y \in \pi (o,\xi _0)$. If $x \rightarrow \xi _0$ then $x \in T_y$ when |*x*| is sufficiently large. Then,

$$\begin{aligned} \left| \frac{f(x)}{\Phi _n(x|\lambda )} - g(\xi _0)\right| = \left| \int _{\partial T} \Bigl (g(\xi )-g(\xi _0)\Bigr )\, \, {\mathrm{d}}\mu _x(\xi )\right| \le 2\Vert g\Vert _{\infty }\, |\mu |_x(\partial T{\setminus } \partial T_y) + \varepsilon \, |{\mathsf m}|_x(\partial T_y). \end{aligned}$$

Now, Lemma 4.2 implies that for $x \rightarrow \xi$ we have $|\mu |_x(\partial T{\setminus } \partial T_y) \rightarrow 0,$ while $|\mu |_x(\partial T_y)$ remains bounded by (17).

Next, we consider a Fatou-type theorem for polyharmonic functions. That is, in the integral of Theorem 4.1 we replace $g(\xi )\, {\mathrm{d}}\xi$ by a complex Borel measure $\nu$ on $\partial T$. We need to consider a restricted type of convergence to the boundary.

### Definition 4.3

Let $\xi \in \partial T$ and $a \ge 0$. The *cone* at $\xi$ of width *a* is

$$\begin{aligned} \Gamma _a(\xi ) = \bigl \{ x \in T : d\bigl (x,\pi (o,\xi )\bigr ) \le a \bigr \}. \end{aligned}$$

The motivation for this definition is well-known: in the open unit disk, consider a cone $C_{\alpha }(z)$ whose vertex is a point *z* on the unit circle, whose axes connects the origin with *z*, and whose opening angle is $\alpha < \pi$. Then, passing to the hyperbolic metric on the disk, all elements of the cone are at bounded distance (depending on $\alpha$) from the axes. The standard graph metric of *T* should be seen as an analogue of the hyperbolic metric on the disk, while a tree-analogue of the Euclidean metric is the ultrametric $\theta$ of (2). Compare with Boiko and Woess [3] for a “dictionary” concerning the many of the other analogies between the potential theory on the unit disk and *T*. Thus, *a* is a substitute for the angle $\alpha$, and of course, if $|x| \rightarrow \infty$ within $\Gamma _a(\xi )$ then $x \rightarrow \xi$ in the topology of $\widehat{T}$. We shall use the following tools.

### **Lemma 4.4**

[11] *For *$$g\in L^1(\partial T,{\mathsf m})$$*, let*

$$\begin{aligned} {\mathcal {M}}g(\xi ) = \sup _{x \in \pi (o,\xi )} \frac{1}{{\mathsf m}(\partial T_x)} \int _{\partial T_x} |g| \, {{\rm d}}{\mathsf m}\end{aligned}$$

*be the associated Hardy–Littlewood maximal function on *$\partial T$. * Then, the operator *$g\mapsto {\mathcal {M}}g$* is weak type (1,1), that is, there is *$C >0$* such that for every *$t > 0$,

$$\begin{aligned} {\mathsf m}\bigl [\, |{\mathcal {M}}g| \ge t\bigr ] \le C\, \Vert g\Vert _1\big /t \quad {\text {for all }}\; g\in L^1(\partial T,{\mathsf m}). \end{aligned}$$

With *R* as in Proposition 3.5, we now define for $a \ge 0$ and $g\in L^1(\partial T,{\mathsf m})$,

18 $$\begin{aligned} {\mathfrak {M}}_a g(\xi ) = \sup \left\{ \left| \int _{\partial T} {\mathcal {K}}_n(x,\cdot |\lambda )\, g\, {{\rm d}}{\mathsf m}\right| : x \in \Gamma _a(\xi ),\; |x| \ge R \right\} . \end{aligned}$$

### Proposition 4.5

*For every *$a \ge 0$*, there is a constant *$C_a$* such that*

$$\begin{aligned} {\mathfrak {M}}_a g\le C_a \,{\mathcal {M}}g\quad {\text {for every }}\; g\in L^1(\partial T, {\mathsf m}). \end{aligned}$$

### *Proof*

Let $\pi (o,\xi )= [o=x_0,x_1,x_2,\ldots ].$ First, let $a = 0$. Fix $x = x_r$ with $r \ge R$. Then, with $A_{\ell } = \{ \eta \in \partial T : |x \wedge \eta | = \ell \}$ as above, we use the properties listed in Lemma 3.2 and compute

$$\begin{aligned} \begin{aligned} \left| \int _{\partial T} {\mathcal {K}}_n(x,\cdot |\lambda )\, g\, {{\rm d}}{\mathsf m}\right|&\le 2 \, \int \limits _{\partial T} \frac{\bigl |K(x,\cdot |\lambda )\, {\mathfrak {h}}(x,\cdot )^n\bigr |}{|\tilde{a}(\lambda )| \,\,|x|^n\,|\widetilde{F}(\lambda )|^{|x|}} \,|g| \, {{\rm d}}{\mathsf m}\\&= \frac{2}{|\tilde{a}(\lambda )|} \,\left| \frac{F(\lambda )}{\widetilde{F}(\lambda )}\right| ^{|x|} \sum _{\ell =0}^{|x|} \int \limits _{A_{\ell }} |F(\lambda )|^{-2\ell }\, \left( \frac{|x|-2\ell }{|x|}\right) ^n |g| \,{{\rm  d}}{\mathsf m}\\&\le \frac{2}{|\tilde{a}(\lambda )|}\,\left| \frac{F(\lambda )}{\widetilde{F}(\lambda )}\right| ^{|x|} \sum _{\ell =0}^{|x|} \,\int \limits _{\partial T_{x_{\ell }}}|F(\lambda )|^{-2\ell }\,|g| \, {{\rm d}}{\mathsf m}\\&\le \frac{2(q+1)}{q\,|\tilde{a}(\lambda )|}\, \sum _{\ell =0}^{|x|} q^{-\ell }|F(\lambda )|^{-2\ell }\,{\mathcal {M}}g(\xi )\\&= \frac{2(q+1)}{q\,|\tilde{a}(\lambda )|}\,\sum _{\ell =0}^{|x|} \left| \frac{F(\lambda )}{\widetilde{F}(\lambda )}\right| ^{|x|-\ell }\,{\mathcal {M}}g(\xi )\\&\le C_0\,{\mathcal {M}}g(\xi ),\qquad {\text {where}} \quad C_0 = \frac{2(q+1)}{q\,|\tilde{a}(\lambda )|}\,\frac{1}{1-|F(\lambda )/\widetilde{F}(\lambda )|}. \end{aligned} \end{aligned}$$

For general $a \in {\mathbb {N}}$, let $y \in T$ with $|y| \ge R$ and $d\bigl (y, \pi (o,\xi )\bigr ) \le a$. Then, $d(x,y) \le 2a$, where *x* is the element on $\pi (o,\xi )$ with $|x|=|y|$. Recall that $|F(\lambda )| <1$. Since $|{\mathfrak {h}}(x,\eta ) - {\mathfrak {h}}(y,\eta )| \le d(x,y)$, we have

$$\begin{aligned} |K(y,\eta |\lambda )| = |F(\lambda )|^{{\mathfrak {h}}(y,\eta )} \le |F(\lambda )|^{-2a} K(x,\eta |\lambda ) \quad {\text {and}}\quad |{\mathfrak {h}}(y,\eta )|^n \le (1+2a)^n |{\mathfrak {h}}(x,\eta )|^n , \end{aligned}$$

for every $\eta \in \partial T$. Therefore,

$$\begin{aligned} |{\mathcal {K}}_n(y,\cdot |\lambda )| \le (1+2a)^n\,|F(\lambda )|^{-2a}\,|{\mathcal {K}}_n(x,\cdot |\lambda )|. \end{aligned}$$

Setting $C_a = (1+2a)^n\,|F(\lambda )|^{-2a}\, C_0\,$, the proposition follows.

After Lemma 4.4 and Proposition 4.5, also the proof of the following theorem now follows the strategy of [11]. For the sake of providing a complete picture in the situation of trees, we also include some of the “standard” details in its proof.

### **Theorem 4.6**

*Let *$\lambda \in {\mathbb {C}}$* with *$|\lambda | > \rho$*, and let *$\nu$* be a complex Borel measure on *$\partial T$*. For *$n \ge 0$*, set*

$$\begin{aligned} f(x) = \int _{\partial T} K(x,\xi |\lambda )\,{\mathfrak {h}}_n(x,\xi |\lambda ) \, {\mathrm{d}}\nu (\xi ). \end{aligned}$$

*Then, f is *$\lambda$*-polyharmonic of order *$n+1$* and*

19 $$\begin{aligned} \lim _{x \rightarrow \xi ,\, x \in \Gamma _a(\xi )} \frac{f(x)}{\Phi _n(x|\lambda )} = g(\xi ) \quad {\text {for every}}\;a \ge 0\; {\text {and }} {\mathsf m}{\text {-almost every }}\; \xi \in \partial T, \end{aligned}$$

*where *$g$* is the Radon–Nikodym derivative of the absolutely continuous part of *$\nu$ *with respect to the uniform distribution *$\mathsf m$* on *$\partial T$.

### *Proof*

We first give an outline of the standard fact that the limit in (19) is 0 when $\nu$ is singular with respect to equidistribution. The latter means that there is a Borel set $E \subset \partial T$ with uniform measure 0 such that $\partial T {\setminus } E$ is a $\nu$-null-set. For every $\varepsilon > 0$, there are disjoint boundary arcs $\partial T_{y_1},\ldots , \partial T_{y_k}$ depending on $\varepsilon$, whose union $E_{\varepsilon }$ contains *E* and has uniform measure $< \varepsilon$. Let $|\nu |$ be the total variation measure of $\nu$. If $x \rightarrow \xi _0 \in \partial T {\setminus } E_{\varepsilon }\,$, then by Lemma 4.2,

$$\begin{aligned} \left| \frac{f(x)}{\Phi _n(x|\lambda )}\right| \le \int _{E_{\varepsilon }} |{\mathcal {K}}_n(x,\xi |\lambda )| \,d|\nu |(\xi ) \rightarrow 0. \end{aligned}$$

Since this holds for every $\varepsilon > 0$, we get that $f(x)/\Phi _n(x|\lambda ) \rightarrow 0$ almost everywhere on $\partial T$.

Now, we may assume without loss of generality that we have $g= {\mathrm{d}}\nu /{{\rm d}}{\mathsf m} m\in L^1(\partial T,{\mathsf m})$. Then, there is a sequence $(g_k)_{k \in {\mathbb {N}}}$ of continuous functions on $\partial T$ such that

$$\begin{aligned} \sum _k \Vert g- g_k \Vert _1 < \infty . \end{aligned}$$

Set

$$\begin{aligned} f_k(x) = \int _{\partial T} K(x,\xi |\lambda )\,{\mathfrak {h}}_n(x,\xi |\lambda )\,g_k(\xi )\,{\mathrm{d}}\xi . \end{aligned}$$

By Lemma 4.4 and Proposition 4.5,

$$\begin{aligned} \sum _k {\mathsf m}\bigl [ {\mathfrak {M}}_a(g-g_k) \ge \varepsilon \bigr ] \le C_a\, C \sum _k \Vert g- g_k \Vert _1/\varepsilon < \infty \end{aligned}$$

for every $\varepsilon > 0$. By the Borel–Cantelli Lemma, this yields that

$$\begin{aligned} {\mathsf m}(A) = 1,\quad {\text {where}}\quad A = \Bigl \{ \xi \in \partial T : \lim _{k \rightarrow \infty } {\mathfrak {M}}_a(g-g_k)(\xi ) = 0 \Bigr \}. \end{aligned}$$

For each *k*, the function on $\widehat{T}$ with values $g_k(\xi )$ for $\xi \in \partial T$ and $f_k(x)/\Phi _n(x|\lambda )$ for $x \in T$ is continuous on $\widehat{T}$ by Theorem 4.1. This readily implies that for $\xi \in A$, we have convergence as proposed in (19).

We now come back to continuous boundary functions and Theorem 4.1. For $n \ge 1$, we cannot expect uniqueness of *f* as a polyharmonic function of order $n+1$ which has the asymptotic behaviour of (15). Indeed, (14) shows that we can add polyharmonic functions of lower order such that the limit in Theorem 4.1 remains the same. However, for the case $n=0$, i.e. for $\lambda$-harmonic functions, we can investigate uniqueness: this case corresponds to the classical Dirichlet problem at infinity. Indeed, for real $\lambda > \rho$ one can use the typical argument, namely the maximum principle, to prove uniqueness. However, for complex $\lambda$, this is not available, and we have to introduce another method.

### **Theorem 4.7**

*Let *$\lambda \in {\mathbb {C}}{\setminus } [-\rho ,\,\rho ]$. * For *$g\in {\mathcal {C}}(\partial T)$*, the function*

$$\begin{aligned} h_{g}(x) = \int _{\partial T} g(\xi )\, K(x,\xi |\lambda )\, {\mathrm{d}}\xi \end{aligned}$$

*is the unique solution of the *$\lambda$*-Dirichlet problem with boundary function *$g$*, i.e. the unique *$\lambda$*-harmonic function such that*

$$\begin{aligned} \lim _{x \rightarrow \xi } \frac{h_{g}(x)}{\Phi (x|\lambda )} = g(\xi ) \quad {\text {for every }}\; \xi \in \partial T. \end{aligned}$$

### *Proof*

Continuity holds by Theorem 4.1. By linearity, we need to prove uniqueness only in the case when $g\equiv 0$. Thus, we assume that $\lambda \cdot h = Ph$ and that $\lim _{|y| \rightarrow \infty } h(y)/\Phi (y|\lambda ) = 0$, and we have to show that $h \equiv 0$.

We extend the notion of the spherical functions as follows:

$$\begin{aligned} \Phi (x,y|\lambda ) = \varphi _{d(y,x)}(\lambda ), \end{aligned}$$

where the functions $\varphi _k$ are given by (9). For fixed $x \in T$, this is the unique $\lambda$-harmonic function of *y* with value 1 at *x* which is radial with respect to the point *x*. Now, let us define the spherical average of *h* around *x*, that is, the function defined by

$$\begin{aligned} \bar{h}(x) = h(x)\quad {\text {and}}\quad \bar{h}(y) = \frac{1}{(q+1)q^{d(y,x)-1}} \sum _{v \,:\, d(v,x) = d(y,x)} h(v), \; {\text { if }}\;y \ne x. \end{aligned}$$

A short computation shows that $\bar{h}$ is $\lambda$-harmonic, whence $\bar{h}(y) = h(x) \Phi (x,y|\lambda )$. By assumption, the function $\widehat{T} \rightarrow {\mathbb {C}}$ with value 0 on $\partial T$ and value $h(y)/\Phi (y|\lambda )$ at $y \in T$ is continuous. By uniform continuity

$$\begin{aligned} \lim _{N \rightarrow \infty } \varepsilon _N = 0, \quad {\text {where}}\quad \varepsilon _N = \sup \{|h(y)/\Phi (y|\lambda )| : y \in T,\; |y| \ge N \}. \end{aligned}$$

Let $y \in T$ be such that $d(y,x) \ge N + |x|$. Then, every $v \in T$ with $d(y,x)=d(v,x)$ satisfies $|v| \ge N$, so that

$$\begin{aligned} |h(v)| \le \varepsilon _N\, |\Phi (v|\lambda )| = \varepsilon _N\, |\Phi (x,v|\lambda )| \,\left| \frac{\Phi (v|\lambda )}{\Phi (x,v|\lambda )}\right| \end{aligned}$$

Applying Proposition 3.5 once more, to both $\Phi (v|\lambda )$ and $\Phi (x,v|\lambda )\,$,

$$\begin{aligned} \frac{\Phi (v|\lambda )}{\Phi (x,v|\lambda )} \sim \widetilde{F}(\lambda )^{|v| - d(v,x)} = \widetilde{F}(\lambda )^{|v| - d(y,x)} \quad {\text {as }}\; |y| \rightarrow \infty . \end{aligned}$$

Since $\widetilde{F}(\lambda )^{|v| - d(v,x)}$ is bounded in absolute value by $\max \{ |\widetilde{F}(\lambda )|^{|x|}, |\widetilde{F}(\lambda )|^{-|x|}\}$, we see that there is a finite upper bound, say $M_x(\lambda )$, depending only on *x* and $\lambda$, such that

$$\begin{aligned} |h(v)| \le \varepsilon _N\, |\Phi (x,v|\lambda )| \,M_x(\lambda ) = \varepsilon _N\, |\Phi (x,y|\lambda )|\,M_x(\lambda ) \quad {\text {whenever }}\; d(v,x)=d(y,x). \end{aligned}$$

Consequently, also the absolute value of the average $\bar{h}(y)$ has the same upper bound. We get

$$\begin{aligned} |h(x)| = \left| \frac{\bar{h}(y)}{\Phi (x,y|\lambda )}\right| \le \varepsilon _N\, M_x(\lambda ). \end{aligned}$$

Letting $N \rightarrow \infty \,$, we conclude that $h(x)=0$, and this holds for any $x \in T$, as required.

Theorem 4.1 tells us that for considering the boundary behaviour of a $\lambda$-polyharmonic function *f* of order *n*, it first should be normalised by dividing by $\Phi ^{(n-1)}(\cdot |\lambda )$.

### **Lemma 4.8**

*Let f be polyharmonic of order n and such that the *$\lambda$*-harmonic function *$h= (\lambda \cdot I - P)^{n-1}f$ *satisfies *

$$\begin{aligned} \lim _{x \rightarrow \xi } \frac{h(x)}{\Phi (x|\lambda )} = g(\xi ) \quad {\text {for all }}\; \xi \in \partial T, \end{aligned}$$

*where *$$g\in {\mathcal {C}}(\partial T)$$*. Then,*

$$\begin{aligned} f(x) = \int _{\partial T} g(\xi )\, K(x,\xi |\lambda )\, {\mathfrak {h}}^{(n-1)}(x,\xi |\lambda )\,d\xi \; + g, \end{aligned}$$

*where gis*$\lambda$*-polyharmonic of order *$n-1$.

### *Proof*

It follows from Theorems 4.7 that

$$\begin{aligned} h(x) = h_{g}(x) = \int _{\partial T} g(\xi )\, K(x,\xi |\lambda )\, {\mathrm{d}}\xi . \end{aligned}$$

Set

$$\begin{aligned} f_{g}(x) = \int _{\partial T} g(\xi )\, K(x,\xi |\lambda )\, {\mathfrak {h}}^{(n-1)}(x,\xi |\lambda )\,{\mathrm{d}}\xi . \end{aligned}$$

By Lemma 3.4,

$$\begin{aligned} (\lambda \cdot I - P)^{n-1} f_{g} = h = (\lambda \cdot I - P)^{n-1} f. \end{aligned}$$

Therefore, $$g= f - f_{g}$$ satisfies $$(\lambda \cdot I - P)^{n-1}g = 0$$.

If in the above lemma, the natural normalisation $g\,\big /\,\Phi ^{(n-2)}(\cdot |\lambda )$ has continuous boundary values, then $g\,\big /\,\Phi ^{(n-1)}(\cdot |\lambda )$ tends to 0 at the boundary of the tree by (14). Thus, by Theorem 4.1, $f\,\big /\,\Phi ^{(n-1)}(\cdot |\lambda )$ has the same boundary limit $g$ as $(\lambda \cdot I - P)^{n-1}f\,\big /\,\Phi (\cdot |\lambda )$.

We conclude that for considering an analogue of the classical Riquier problem, with given boundary functions $g_0,\ldots , g_{n-1}\,$, our solution *f* should be obtained step-wise: first, $(\lambda \cdot I - P)^{n-1}f\,\big /\,\Phi (\cdot |\lambda )$ should have boundary limit $g_{n-1}\,$, and we take $f_{n-1}=f_{g_{n-1}}$ according to Lemma 4.8. Next, the function $f - f_{n-1}$ should be polyharmonic of order $n-1$, and $(\lambda \cdot I - P)^{n-2}(f - f_{n-1})\,\big /\,\Phi (\cdot |\lambda )$ should have boundary limit $g_{n-2}$. We then proceed recursively. We clarify this by the next definition.

### Definition 4.9

Let $\lambda \in {\mathbb {C}}{\setminus } [-\rho ,\,\rho ]$ and $g_0, \ldots , g_{n-1} \in {\mathcal {C}}(\partial T)$. Then, a solution of the associated Riquier problem at infinity is a polyharmonic function

$$\begin{aligned} f = f_0 + \cdots + f_{n-1} \end{aligned}$$

of order *n*, where each $f_k$ is polyharmonic of order $k+1$ and

$$\begin{aligned} \lim _{x \rightarrow \xi } \frac{(\lambda \cdot I - P)^k f_k(x)}{\Phi (x|\lambda )} = g_k(\xi ) \quad {\text {for every }}\; \xi \in \partial T . \end{aligned}$$

### **Corollary 4.10**

*A solution of the Riquier problem as stated in Definition *4.9* is given by the functions*

$$\begin{aligned} f_k(x) = \int _{\partial T} g_k(\xi )\, K(x,\xi |\lambda ) \,{\mathfrak {h}}_k(x,\xi |\lambda )\, {\mathrm{d}}\xi . \end{aligned}$$

*One also has*

$$\begin{aligned} \lim _{x \rightarrow \xi } \frac{f_0(x)+ \cdots + f_k(x)}{\Phi _k(x|\lambda )} = \lim _{x \rightarrow \xi } \frac{f_k(x)}{\Phi _k(x|\lambda )} = g_k(\xi ) \quad {\text {for every }}\; \xi \in \partial T. \end{aligned}$$

As already outlined further above, the solution is *not* unique. We can add to $f_k$ some suitable $\lambda$-polyharmonic function of lower order: normalised by $\Phi _k(x|\lambda )$, by (14) the latter will tend to zero, as $|x| \rightarrow \infty$. What is unique is—by Theorem 4.7—the solution $(\lambda \cdot I - P)^k f_k = h_{g_k}\,$.
